# Advancing the diagnosis and treatment of posterior ligamentous complex inflammatory syndrome (PLCIS) in chronic axial low back pain

**DOI:** 10.1016/j.inpm.2025.100629

**Published:** 2025-08-21

**Authors:** Javier De Andrés Ares

**Affiliations:** Hospital Universitario La Paz, Madrid, Spain

Over the past three decades, considerable progress has been made in understanding the anatomical origins of spinal pain, prompting a critical reassessment of what was once broadly labeled as “nonspecific low back pain.” As diagnostic precision has improved, it has become increasingly evident that specific pain generators can be isolated and treated with interventional techniques. The zygapophyseal joints have emerged as well-established sources of axial lumbar pain [1]. Their role can be reliably confirmed via diagnostic medial branch blocks, followed by medial branch radiofrequency neurotomy, which has demonstrated consistent therapeutic benefit. [2,3]. Similarly, the sacroiliac joint and the posterior sacroiliac ligamentous complex have been implicated in gluteal and lower back pain and are amenable to treatment using image-guided blocks and radiofrequency ablation [4,5]. Also recently published in Interventional Pain Management superior cluneal nerve entrapment has also emphasized to be an underdiagnosed cause of localized gluteal pain [6]. Moreover, while the intervertebral disc has long been regarded as a potential nociceptive source, effective long-term treatment options for discogenic pain remain limited [7,8].

Despite these advancements, a portion of chronic low back pain continues to elude precise etiological categorization. However, the ongoing refinement of anatomical knowledge and imaging modalities is progressively narrowing that uncertainty.

A significant development in the last decade is the identification of a novel clinical-radiological condition: Posterior Ligamentous Complex Inflammatory Syndrome (PLCIS) [9]. PLCIS is characterized by a constellation of findings—including variable fluid signal intensity, contrast enhancement, and local inflammatory changes—centered within the retrodural space of Okada (RSO). This space, an extradural anatomical compartment located dorsal to the ligamentum flavum, has traditionally been underrecognized but offers meaningful insights into previously unexplained presentations of axial and radicular pain [10]. Originally described by Kikuzo Okada in 1981 through cervical facet joint arthrography, the RSO forms a functional communication channel linking facet joints, interspinous spaces, and surrounding structures [11]. This space allows for the spread of inflamation, fluid, and contrast material between compartmental elements of the posterior ligamentous complex—including facet joints, interspinous bursae (present in Baastrup disease), pars interarticularis defects, and paraspinal tissues [[12], [13], [14]]. It is often visualized as a channel for contrast during fluoroscopic or CT guided injections, commonly showing characteristic patterns such as butterfly- or ovoid-shaped flows in the presence of pathological communication [15,16]. The RSO's role in pain syndromes is increasingly recognized. It serves as a central nexus in the PLCIS, a multi compartment process driven by fluid and inflammation spreading via RSO [9]. Typical imaging findings can include T2 hyperintensity, contrast enhancement, and the co-occurrence of changes in paravertebral structures. These abnormalities may correlate with axial low back pain and radiculopathy, but require careful clinical correlation [10]. Notably, the formation of interspinous adventitial bursae (Baastrup disease) and synovial cysts, often seen in degenerative or unstable segments, may relate to pathological communication through the RSO [9].

The article by Shah and Vorobeychik introduces a critical advancement in addressing one enigmatic source of axial LBP: PLCIS [17]. By presenting a detailed case and highlighting novel interventional strategies, the authors foster a necessary dialogue on how to better identify and treat this under-recognized syndrome. The article describes a compelling case wherein a patient with persistent, severe axial low back pain failed to respond to medical and physical therapies. Standard anteroposterior and dynamic radiographs revealed only non-specific degenerative changes. Crucially, a SPECT scan demonstrated intense radiotracer uptake both in the bilateral L4-L5 facet joints and the L4-L5 interspinous ligament—findings indicative of PLCIS. The pivotal therapeutic step involved injecting steroid solution into the L4-L5 adventitial interspinous bursa. Imaging with contrast confirmed that the medication spread through the space of Okada into the adjoining compartments, including the facet joints. This approach led to impressive clinical improvement. This case highlights several critical points that may broaden the management strategies for axial back pain: SPECT scan is valuable and more accessible for the diagnosis of PLCIS, particularly when MRI is contraindicated or denied by payers. Also therapeutic injection of local anesthetics and corticosteroids into the interspinous bursa, can offer significant and lasting relief.

Shah and Vorobeychik's article broadens the diagnostic and therapeutic toolbox for chronic axial low back pain by illuminating the role of PLCIS. Their case provides a rationale for considering PLCIS and utilizing targeted bursal injections in appropriately selected patients. In the ongoing pursuit of precise, effective pain management, such careful clinical observation and intervention offer hope for those suffering from the burdens of persistent spinal pain.

## Declaration of competing interest

The author declares to have no known competing financial interests or personal relationships that could have appeared to influence the work reported in this paper.
